# Investigating the Effects of Gardenia Polysaccharides on LPS-Induced Immune Injury in Mice and Exploring the Molecular Mechanisms Underlying Its Regulatory Effect on the Immune Function of Macrophages

**DOI:** 10.3390/foods14203455

**Published:** 2025-10-10

**Authors:** Pingdong Lin, Wen Yue, Han Xiang, Jing Liu, Xinzhu Chen

**Affiliations:** 1 Institute of Animal Husbandry and Veterinary Medicine, Fujian Academy of Agricultural Sciences, Fuzhou 350013, China; lpd7911@163.com (P.L.); yuewen202301@163.com (W.Y.); fjliuj@163.com (J.L.); 2 Fujian Key Laboratory of Animal Genetics and Breeding, Fuzhou 350013, China; 3 College of Life Sciences, Fujian Normal University, Fuzhou 350117, China; 17705078951@163.com

**Keywords:** *Gardenia jasminoides* Ellis, polysaccharides, TLR4, NF-κB, immunomodulation, oxidative stress, macrophage

## Abstract

This study investigated the protective effects of *Gardenia jasminoides* Ellis polysaccharides (GP) on lipopolysaccharide (LPS)-induced immunosuppression and oxidative stress in mice and explored how GP modulates macrophage polarization through the TLR4/NF-κB signaling axis. The results showed that GP notably restored thymus and spleen indices in LPS-treated mice, markedly decreased the serum concentrations of malondialdehyde, and enhanced superoxide dismutase activity and total antioxidant capacity. In RAW 264.7 macrophage cultures, GP displayed immunostimulatory effects by improving phagocytic activity, promoting NO synthesis, and enhancing the secretion of pro-inflammatory cytokines, including IL-1β, IL-6, and TNF-α. These effects were observed in cells not pretreated with TAK-242 or PDTC; however, they were not observed in cells pretreated with these inhibitors. At 300 µg/mL concentration, GP markedly enhanced the transcriptional levels of iNOS and cytokine genes. Protein analysis revealed significant upregulation of TLR4, MyD88, TRAF6, NF-κB RelA/p65, and phosphorylated p65. Fluorescence imaging confirmed the nuclear translocation of p65. Collectively, these findings indicated that GP reversed systemic immunosuppression and oxidative stress, offering foundational insights for developing natural immune regulators. The observed immunomodulatory properties of GP are likely mediated through the TLR4/NF-κB signaling pathway.

## 1. Introduction

Lipopolysaccharide (LPS) potently activates the nuclear transcription factor kappa B (NF-κB) signaling axis via toll-like receptor 4 (TLR4), driving macrophage polarization toward the M1 phenotype. This activation induces the release of pro-inflammatory factors and triggers severe oxidative stress, culminating in systemic immunosuppression. Molecularly, this process relies on the myeloid differentiation primary response gene 88 (MyD88)-dependent pathway, characterized by a marked upregulation of critical inflammatory markers, including interleukin (IL)-1β and tumor necrosis factor (TNF)-α. These elements interact with reactive oxygen species (ROS) accumulation after nicotinamide adenine dinucleotide phosphate (NADPH) oxidase activation, forming a self-perpetuating inflammation-oxidative stress loop [1].

Polysaccharides are natural metabolites found in plants, animals, and microorganisms, and possess a wide range of biological properties, including immunomodulatory, anti-tumor, anti-aging, anti-inflammatory, antioxidant, hypoglycemic, and hypolipidemic properties [2,3,4]. Immunomodulation is considered one of the most prominent characteristics of polysaccharides. Although the immunomodulatory properties of natural polysaccharides, such as *Astragalus* and *Ganoderma*, have been well elucidated [5,6], *Gardenia* polysaccharides (GP) remain largely unexplored.

GP is an acidic pectin polysaccharide derived from the dried fruit of *Gardenia jasminoides* Ellis. It has a molecular weight (Mw) of approximately 45 kDa. Although previous studies have shown the immunomodulatory potential of GP [7], its precise molecular mechanisms remain poorly characterized. Notably, it has been reported that β-glucan can regulate the immune system through the dectin-1 receptor [8], and the unique C6 acetylation modification structure of GP may form a unique spatial conformation, endowing it with differentiated biological characteristics.

The immune system comprises both innate and adaptive immunity, which are collaboratively involved in immune defense, surveillance, and regulation. Immune organs, including the spleen and thymus, and cellular components, such as macrophages and natural killer cells, are involved in these functions [9]. The effect of polysaccharides on the immune system has been widely studied, revealing a complex and multifactorial process that involves various signaling pathways and cellular mechanisms [10]. Innate immune cells, which mediate non-specific immune responses, play a critical role in these processes [5].

Macrophages, key players in innate immunity, are actively involved in immunomodulation through processes such as chemotaxis, phagocytosis, and direct cytotoxicity against pathogens. These cells can be activated by polysaccharides to combat pathogens and modulate immune responses through two major mechanisms, endocytosis-mediated activation and receptor-mediated activation, with the latter playing a dominant role [11,12]. Such complex carbohydrate molecules frequently interact with surface-expressed pattern-recognition receptors (PRRs), such as carbohydrate-conjugated signaling molecules, thereby triggering cellular immune response and driving differentiation toward the M1 phenotype (pro-inflammatory state) [13]. Activated M1 macrophages release reactive nitrogen intermediates, like nitric oxide (NO), along with inflammatory mediators, such as TNF-α, IL-1β, and IL-6. These cytokines initiate immune response, enhance phagocytic activity, and promote the immunomodulatory effects of macrophages [14,15].

Studies have shown that toll-like receptors (TLRs) play pivotal roles in polysaccharide recognition by macrophages, with TLR4 being a critical receptor for plant-derived polysaccharides [16,17,18]. Polysaccharides function as ligands, which bind to TLR4 and transmit extracellular signals to target cells through the TLR4-CD14 complex, thereby triggering a series of intracellular signaling cascades. This process leads to NF-κB pathway activation and cytokine release [19,20]. Macrophage stimulation by polysaccharides represents an essential process for modulating innate immunity. In line with our previous findings, GP can activate macrophages, suggesting its immunoregulatory properties [7].

This study aimed to systematically elucidate the role of GP in terms of both immunomodulation and antioxidant defense and uncover its effect on macrophages polarization.

## 2. Materials and Methods

### 2.1. Materials and Reagents

GP (Mw ≈ 45 kDa, acidic pectin/galacturonic acid-rich domains [7]) was extracted from dried *Gardenia jasminoides* fruits via the hot water extraction method. Thereafter, ethanol precipitation (80% *v*/*v*) and decolorization were conducted using macroporous resin, and deproteinization was conducted using the repeated freezing-thawing method. The superoxide dismutase (SOD, HY-M0001), malondialdehyde (MDA, HY-M0003), and total antioxidant capacity (T-AOC, HY-M0011) detection kits were purchased from Beijing Sino-Uk Institute of Biological Technology (Beijing, China). RAW 264.7 cell culture medium (CM-0190) was purchased from Wuhan Pricella Biotechnology Co., Ltd. (Wuhan, China). Shanghai Beyotime Biotechnology provided multiple assay kits, including enhanced CCK-8, neutral red detection, nitric oxide measurement, and NF-κB nuclear translocation analysis kits. Beijing 4A Biotech provided ELISA detection kits for cytokines, and Wuhan Igenebook Biotechnology synthesized all specific primers.

Molecular biology reagents, including RNA isolation kits, Taq polymerase, cDNA synthesis kits, and SYBR^®^ Premix Ex Taq™, were purchased from Hangzhou Simgen Biotechnology (Hangzhou, China). TLR4 polyclonal antibody (#19811-1-AP) was purchased from Wuhan Sanying Biotechnology (Wuhan, China). Antibodies against phospho-NF-κB p65 (#3033), MyD88 (#4283), NF-κB p65 (#8242), and TRAF6 (#67591) were purchased from Cell Signaling Technology (BOS, Danvers, MA, USA). Quality control antibodies included β-actin (#T0022) from Shanghai Affinity Biopharmaceutical (Shanghai, China) and Histone 3 (#ab1791) from Abcam (Cambridge, UK). Pharmacological inhibitors resatorvid (TAK-242) and PDTC were purchased from MedChemExpress (Monmouth Junction, NJ, USA), and cellular protein extraction reagents were purchased from Beijing Solarbio Science & Technology (Beijing, China).

### 2.2. Laboratory Animals and Grouping

In total, 30 male Kunming (KM) mice (SPF grade, aged 6 weeks, body weight 20.0 ± 2.0 g) were procured from Shanghai Shengchang Biotechnology Co., Ltd. (Shanghai, China). The breeding conditions for mice were as follows: temperature maintained at 25 ± 1 °C, relative humidity controlled between 70% and 75%, a daily light exposure of 12 h, and ad libitum access to drinking water. The animal experiments were approved by the Laboratory Animal Ethical Committee of Animal Husbandry and Veterinary Medicine, Fujian Academy of Agricultural Sciences (Approval No.: 202402GJ009). Following a 7-day acclimatization period, the mice were randomly divided into three experimental groups (*n* = 10 per group): therapeutic intervention (GP) group, disease model (MC) group, and healthy control (NC) group. Throughout the 14-day treatment period, animals in the GP group received oral gavage of the GP solution (250 mg/kg/day) based on data from a pilot study. Animals in both the NC and MC groups received equivalent volumes of physiological saline. One hour after the final oral gavage, mice in the MC and GP groups intraperitoneally received LPS (2 mg/kg) to induce oxidative stress and immunosuppression. Concurrently, mice in the NC group received an equivalent volume of normal saline via intraperitoneal injection. Subsequently, all mice were subjected to a 12 h fasting period during which water was available ad libitum. Following the fasting period, mice were weighed, and venous blood samples and tissues, including the spleen, thymus, and intestinal tissues, were collected for further analysis.

#### 2.2.1. Calculation of Immune Organ Index

Body weights of the mice were recorded before euthanasia. Subsequently, the thymus and spleen were carefully excised, and all surrounding connective and adipose tissues were carefully removed before precise weighing. The immune organ index was subsequently determined using the following formula:

(1) $$\mathrm{Immune}\;\mathrm{organ}\;\mathrm{index}\;=\;\frac{\mathrm{immune}\;\mathrm{organ}\;\mathrm{weight}\;(\mathrm{mg})}{\mathrm{body}\;\mathrm{weight}\;(g)}$$

#### 2.2.2. Pathological Examination of Intestinal Mucosal

Small intestine specimens were meticulously fixed in 4% paraformaldehyde solution. Following fixation, the specimens underwent embedding procedures and were thinly sliced using precise techniques. Hematoxylin-eosin (HE) staining, a standard histopathological method, was subsequently applied to these sections. Tissue sections were scanned and imaged using the PANNORAMIC panoramic slide scanner (3DHISTECH, Ltd., Budapest, Hungary). The target area of the intestinal tissue was selected using CaseViewer 2.4 software. Following image acquisition, Image-Pro Plus (IPP) 6.0 software was employed to measure the villus height, crypt depth, and mucosal layer thickness. The mean value of five measurements for each parameter was calculated and used as the final result.

#### 2.2.3. Evaluation of the Serum Biomarkers of Oxidative Stress

Blood samples from all experimental groups were obtained via retro-orbital bleeding immediately before cervical dislocation. Following collection, the samples underwent centrifugation at 4 °C (3000 rpm, 10 min) for serum isolation. The harvested serum sample was aliquoted into single-use portions at 4 °C and cryopreserved at −80 °C until further analysis. Oxidative stress biomarkers, including SOD, MDA, and T-AOC, were quantified through colorimetric assays strictly following standardized protocols.

### 2.3. Cell Culture and Experimental Design

The RAW 264.7 cell line (mouse leukemic monocyte/macrophage line) was purchased from the American Type Culture Collection (ATCC), Manassas, VA, USA. These cells were cultured in Dulbecco’s Modified Eagle Medium (DMEM) containing 10% fetal bovine serum and 1% penicillin-streptomycin solution. Incubation was conducted at 37 °C in a humidified environment with 5% CO_2_ concentration. Routine subculturing procedures were conducted at 2–3-day intervals [21].

The viability of RAW 264.7 macrophages after GP exposure was evaluated via the CCK-8 assay. Initially, macrophage suspensions (5 × 10^4^ cells/mL density) were plated into 96-well culture dishes (100 μL per well) and maintained under standard culture conditions until complete adhesion. Subsequently, different concentrations of GP (75–1200 μg/mL) were added to the culture plates, which were then divided into two parts and incubated for 24 h and 48 h, respectively. Thereafter, CCK-8 reagent was administered (10 μL per well) for 1 h of incubation. Optical density was read at 450 nm wavelength using an ELISA plate analyzer (SYNERGY-H1, Flash Spectrum Biotechnology Co., Ltd., Shanghai, China) to quantify cellular metabolic activity.

RAW 264.7 cells were categorized into three experimental conditions: In the inhibitor-treated cohort, cellular samples underwent 30 min pre-incubation with either TAK-242 (25 µM concentration) or PDTC (50 µM concentration) before exposure to GP. The GP group received only GP without prior treatment with an inhibitor. The control group consisted of cells, which did not receive GP or inhibitor treatment.

#### 2.3.1. Cell Phagocytosis Assay

Cellular phagocytic activity was measured through neutral red phagocytosis experiments. RAW 264.7 cells in optimal condition were treated based on the predefined grouping protocol. A 0.1% neutral red staining solution (100 μL per well) was introduced into each well, with subsequent incubation lasting 60 min. After incubation, cellular supernatants were discarded, and three PBS washes were performed to remove residual dye. Cellular lysis was induced by adding a lysing agent composed of ethanol and acetic acid in equal proportions (100 μL per well). The absorbance was read at 540 nm after incubation at room temperature for 2 h.

#### 2.3.2. Measurements of NO and Cytokine Concentrations in Cell Supernatants

RAW 264.7 cells were seeded in 6-well plates. Following pretreatment with the inhibitor (TAK-242/PDTC) and GP, the cell supernatant was harvested to quantify NO, IL-1β, IL-6, and TNF-α levels. The measurements were conducted following the manufacturer’s instructions.

#### 2.3.3. Quantitative Analysis of iNOS and Cytokine mRNA Expression

The mRNA levels of iNOS, IL-1β, IL-6, and TNF-α were measured via real-time quantitative PCR (qPCR) technology. Total RNA was isolated from RAW 264.7 cells using the Trizol method, with purity and quantity verification conducted using a NanoDrop™ spectrophotometer (Thermo Fisher NanoDrop™ One/OneC, Waltham, MA, USA). A commercial cDNA synthesis kit was employed to reverse transcribe RNA into cDNA, following the manufacturer’s instruction. Specific primers and fluorescent probes were designed using Primer 5.0 software based on target gene sequences and verified using Oligo7 and Prime-blast software (https://biodb.swu.edu.cn/qprimerdb/). Primer specificity was confirmed through electrophoretic separation on agarose matrices. Amplification reactions were conducted on a 7500-qPCR platform (Thermo Fisher) using SYBR^®^ Premix Ex Taq™ chemistry. Gene expression level was normalized against the housekeeping gene β-actin, and fold-change calculations were conducted through comparative threshold cycle (2^−ΔΔCt^) analysis methodology. The primer information utilized in the qPCR experiment is presented in Table 1.

#### 2.3.4. Western Blotting

The expression of TLR4, MyD88, TRAF6, p65, and p-p65 proteins was determined through immunoblotting. Both whole-cell lysates and nuclear extracts of RAW 264.7 cells were prepared, with protein levels measured using the BCA assay method. Protein samples underwent separation through SDS-PAGE before being transferred to PVDF membranes via a semi-dry transfer system. The membranes were blocked with 5% non-fat milk in TBST buffer for 120 min at ambient temperature (phosphorylated targets required BSA-based blocking solution). Overnight incubation was performed at 4 °C, with primary antibodies diluted in TBST. Thereafter, three TBST washing cycles were conducted. Corresponding secondary antibodies prepared under identical dilutions were subsequently applied for immunodetection. After removing unbound secondary antibodies through repeated TBST rinses, the chemiluminescence reaction was conducted using an enhanced chemiluminescence (ECL) reagent. Finally, the grayscale values of the target band were analyzed using IPP 6.0. The densitometric values of target protein bands were normalized to those of the corresponding loading control band (β-actin) in the same lane. Data were obtained from three independent biological replicates. The experiments were conducted on cells cultured from different passages.

#### 2.3.5. Immunofluorescence Analysis

RAW 264.7 cells were plated in 12-well culture dishes precoated with sterile coverslips (φ20 mm) and exposed to inhibitors and/or GP for 24 h. The cells were fixed with fixative solution for 15 min following culture medium removal and three PBS washes. Subsequently, an immunofluorescence reagent was used to block the fixed cells. One hour later, the cells were incubated with NF-κB p65-specific primary antibody at 4 °C overnight. Unbound antibodies were eliminated through PBS rinsing. Next, the samples were incubated with fluorescence-conjugated secondary antibodies at room temperature for 60 min. After repeating the PBS washing, cell nuclei were counterstained using DAPI solution. Finally, the staining solution was removed, and the slide was sealed. The coverslips were observed using a Leica DMIL-LED fluorescence microscope. The positive rates of red fluorescence signals (RFS, indicating p65 expression) in the whole-cell, cell membrane, and cell nucleus were quantified using Aipathwell^®^ (V2.1.3) software. Cytoplasmic RFS was obtained by subtraction of whole-cell and cell membrane. Subsequently, the nuclear/cytoplasmic fluorescence ratio was calculated.

### 2.4. Statistical Analysis

We analyzed the average values obtained from three separate experimental trials. Quantitative data are expressed as mean ± standard deviation and visualized as bar charts generated using GraphPad Prism 8. Statistical significance was determined using one-way analysis of variance (ANOVA) followed by Tukey’s multiple comparisons test in SPSS 20.0. A predetermined threshold of α = 0.05 established statistical significance, with probability values below this critical level (*p* < 0.05) suggesting a meaningful difference between groups.

## 3. Results

### 3.1. Effects of GP on the Immune Function of Mice

**Figure 1:**
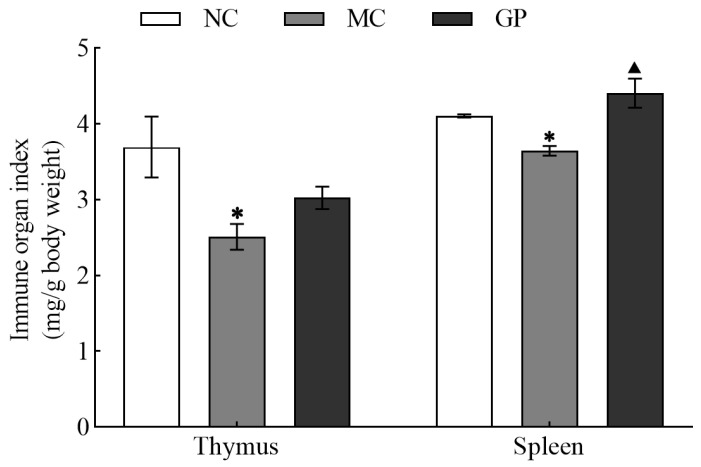
The effect of GP on thymic and splenic indices in mice. Experimental results are displayed as mean values ± standard deviation (n = 10). Symbols (* or ▲) marked above the bars indicate significant differences (*p* < 0.05). * denotes *p* < 0.05 compared to the NC group and ▲ represents *p* < 0.05 compared to the MC group.

As illustrated in Figure 1, the MC group showed markedly reduced thymus and spleen indices compared to the NC group (*p* < 0.05). Importantly, GP significantly increased the spleen index compared to the MC group (*p* < 0.05), but did not significantly affect thymus recovery. Together, these data indicated that GP effectively reversed the LPS-induced suppression of immune organ indices in mice.

### 3.2. Effects of GP on the Morphological Characteristics of Intestinal Mucosa in Mice

**Figure 2:**
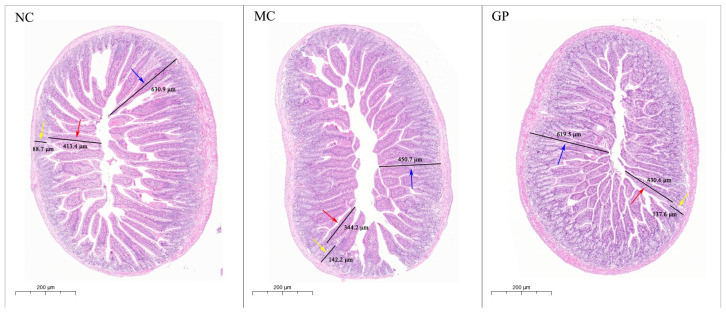
The effect of GP on the morphological characteristics of intestinal mucosa in mice (HE 5.0×). The standard unit of measurement was micrometer (μm). The red arrow denotes villus height, the yellow arrow denotes crypt depth, and the blue arrow denotes mucosal layer thickness.

HE staining of murine intestinal mucosa is presented in Figure 2. In the NC group, intestinal villi appeared intact and neatly aligned, and crypt structures exhibited normal morphology. In contrast, mice treated with LPS alone (MC group) showed pathological alterations in the intestinal mucosa. Quantitative analysis (Table 2) revealed that villus height and mucosal layer thickness were significantly decreased in the MC group compared to the NC group (*p* < 0.05), along with significantly increased crypt depth (*p* < 0.05) and a significantly reduced villus-to-crypt ratio (V/C, *p* < 0.05). These findings collectively indicate structural damage to the intestinal mucosa. GP significantly restored villus height, mucosal thickness, and V/C values compared to the MC group (*p* < 0.05), suggesting that GP can effectively alleviate LPS-induced intestinal mucosal injury.

### 3.3. The Effects of GP on the Serum Levels of Oxidative Stress Biomarkers in Mice

**Figure 3:**
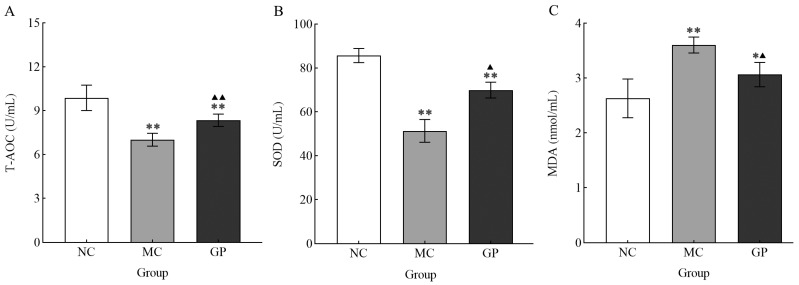
The effect of GP on the serum level T-AOC (**A**), SOD (**B**), and MDA (**C**) in mice. Experimental outcomes are presented as mean values ± standard deviation (*n* = 10 per group). Data points marked with distinct superscript symbols (* and ▲) denote significant differences (*p* < 0.05). Asterisks indicate comparisons with the NC group (* *p* < 0.05, ** *p* < 0.01), while upward-pointing triangles represent differences compared to the MC group (▲ *p* < 0.05, ▲▲ *p* < 0.01).

Serum analysis showed significant differences in oxidative stress markers between the experimental groups (Figure 3). Mice in the MC group showed significantly decreased SOD and T-AOC activities compared to the NC group (*p* < 0.05), coupled with a notable rise in MDA concentration (*p* < 0.05). In contrast, mice in the experimental group pre-treated with GP exhibited a significant increase in the serum activity of SOD and T-AOC (*p* < 0.05), alongside a marked decline in MDA levels (*p* < 0.05). These findings indicated that GP mitigated oxidative stress after LPS stimulation, suggesting its antioxidant property.

### 3.4. The Effects of GP on Cell Survival and Phagocytic Function

**Figure 4:**
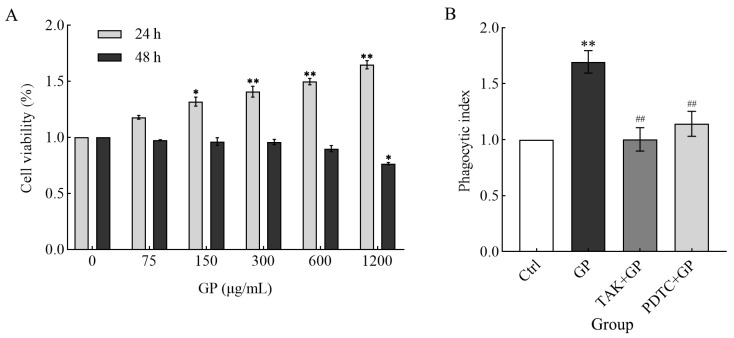
(**A**) The effect of different concentrations of GP (0 μg/mL as control, 75, 150, 300, 600, and 1200 μg/mL) on RAW264.7 cell survival over two incubation periods (24 and 48 h). (**B**) The effects of GP on the phagocytic activity of RAW264.7 cells. The groups comprised control medium (Ctrl), GP (300 µg/mL), TAK-242 (25 µmol/L) + GP (300 µg/mL), and PDTC (50 µmol/L) + GP (300 µg/mL). Statistical significance markers (* *p* < 0.05, ** *p* < 0.01) indicate differences compared to the Ctrl group, while hash symbols (## *p* < 0.01) denote comparisons between the inhibitor group (TAK + GP group or PDTC + GP group) and the GP group.

Compared to the control group, GP markedly increased the survival rates of RAW 264.7 cells after 24 h incubation (concentration range: 75–1200 μg/mL; *p* < 0.05, *p* < 0.01) (Figure 4A). In contrast, extended exposure over 48 h revealed divergent outcomes: concentrations between 75 and 600 μg/mL showed minimal effect on cell viability, while the highest tested dose (1200 μg/mL) induced a substantial decline in viable cell counts. Based on these results, 24 h treatment with 300 µg/mL GP was selected as the optimal condition for subsequent experiments to maintain favorable cellular viability.

RAW 264.7 cells treated with GP exhibited markedly enhanced phagocytic capacity compared to untreated control cells (*p* < 0.01). On the contrary, the phagocytic activity of the TAK-242 + GP and PDTC + GP groups was suppressed to the control level (Figure 4B), suggesting that the TLR4/NF-κB signaling is indispensable for GP-induced phagocytosis. These findings suggest that GP enhanced phagocytosis in RAW 264.7 cells, potentially by activating TLR4 receptors on the cell surface, which in turn triggers the downstream NF-κB signaling pathway.

### 3.5. The Effect of GP on Nitric Oxide Synthesis and iNOS mRNA Expression in Cells

**Figure 5:**
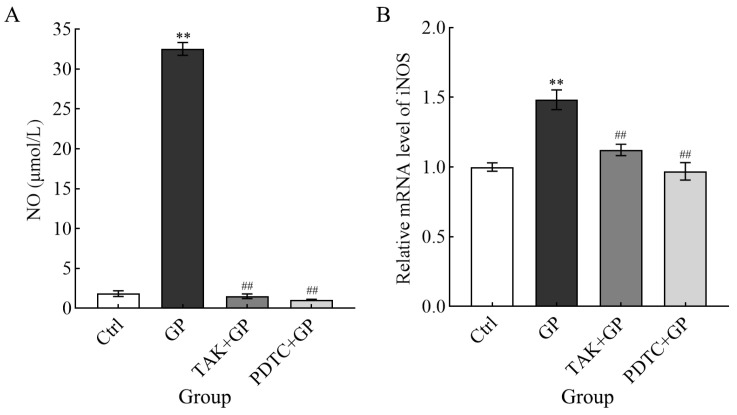
The effect of GP on NO generation (**A**) and the level of iNOS mRNA (**B**) in RAW264.7 macrophages. Analysis of the mRNA levels of the target genes was conducted compared to the housekeeping gene β-actin. Cellular treatments comprised control medium (Ctrl), GP (300 µg/mL), TAK-242 (25 µmol/L) + GP (300 µg/mL), and PDTC (50 µmol/L) + GP (300 µg/mL). ** indicates *p* < 0.01 versus the Ctrl group, while ## signifies *p* < 0.01 for the inhibitor group (TAK + GP group or PDTC + GP group) compared to the GP group.

RAW 264.7 cells were treated with GP and exhibited a marked increase in NO levels compared to the control group (*p* < 0.01) (Figure 5A). In contrast, both TAK + GP and PDTC + GP co-treatment groups revealed markedly reduced NO generation compared to GP alone (*p* < 0.01), suggesting the role of GP in stimulating NO synthesis in these cells. Additionally, GP significantly upregulated the mRNA expression of iNOS compared to untreated controls (*p* < 0.01). There was no significant difference between the inhibitor combination group and the control group in terms of iNOS mRNA levels. Nevertheless, both inhibitor-treated groups showed considerably lower expression than GP-treated cells (*p* < 0.01, Figure 5B). These data suggest that GP enhanced NO release in RAW 264.7 macrophages through transcriptional activation of iNOS.

### 3.6. The Effects of GP on Cytokine Secretion and Gene Expression at the Transcriptional Level

**Figure 6:**
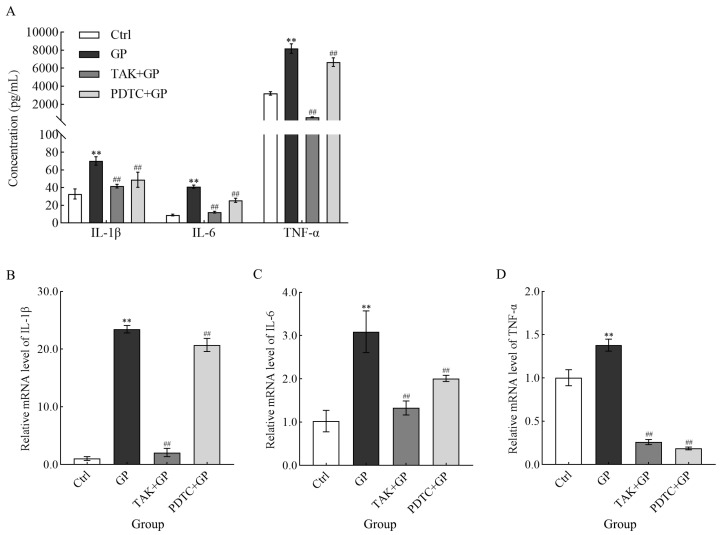
The effect of GP on IL-1β, IL-6, and TNF-α secretion (**A**), along with the mRNA expression levels of IL-1β (**B**), IL-6 (**C**), and TNF-α (**D**) in RAW264.7 macrophages. The mRNA levels of target genes were quantified using β-actin as the internal reference. Cellular treatments comprised control medium (Ctrl), GP (300 µg/mL), TAK-242 (25 µmol/L) + GP (300 µg/mL), and PDTC (50 µmol/L) + GP (300 µg/mL). ** indicates *p* < 0.01 versus the Ctrl group, while ## signifies *p* < 0.01 for the inhibitor group (TAK + GP group or PDTC + GP group) compared to the GP group.

In this study, the release of pro-inflammatory mediators IL-1β, IL-6, and TNF-α from RAW 264.7 cells exposed to GP (300 μg/mL) showed a notable increase compared to the untreated control (*p* < 0.01) (Figure 6A). However, cells pretreated with either TAK-242 (the TLR4 inhibitor) or PDTC (the NF-κB inhibitor) secreted markedly lower levels of these cytokines compared to cells stimulated with GP alone (*p* < 0.01). This suggested that GP potentiated the production of inflammatory mediators in macrophages, potentially by activating the TLR4/NF-κB pathway. Parallel transcriptional analyses revealed significantly elevated mRNA expression levels of IL-1β, IL-6, and TNF-α in GP-treated cells compared to control cells (*p* < 0.01). Conversely, in cells pretreated with inhibitors, the mRNA levels of cytokines were markedly decreased after treatment with GP (*p* < 0.01) (Figure 6B). This further confirms the aforementioned results at the genetic level.

### 3.7. GP Stimulated the TLR4/NF-κB Signaling Cascade

**Figure 7:**
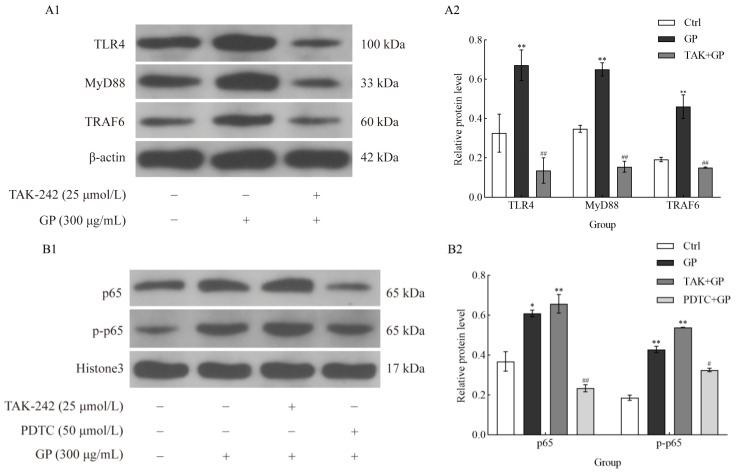
The effect of GP on the protein expression levels of TLR4, MyD88, TRAF6, p-p65, and p65 in RAW264.7 macrophages. (**A1**) Immunoblotting bands of TLR4, MyD88, TRAF6, and β-actin (as internal reference). (**A2**) Quantitative of TLR4, MyD88, and TRAF6. Cellular treatments comprised control medium (Ctrl), GP (300 µg/mL), and TAK-242 (25 µmol/L) + GP (300 µg/mL). (**B1**) Immunoblotting bands of p65, p-p65, and Histone 3 (as internal reference). (**B2**) Quantitative analysis of p65 and p-p65 levels. Cellular treatments comprised control medium (Ctrl), GP (300 µg/mL), TAK-242 (25 µmol/L) + GP (300 µg/mL), and PDTC (50 µmol/L) + GP (300 µg/mL). Data represent three independent experiments. Statistical significance markers (* *p* < 0.05, ** *p* < 0.01) indicate differences compared to the Ctrl group, while hash symbols (# *p* < 0.05, ## *p* < 0.01) denote comparisons between the inhibitor group (TAK + GP group or PDTC + GP group) and the GP group.

Western blotting (Figure 7A1,A2) indicated that GP substantially upregulated the critical components of the TLR4 signaling pathway in RAW 264.7 macrophages, specifically TLR4, MyD88, and TRAF6 (*p* < 0.01). We used the TLR4 inhibitor TAK-242 to confirm the role of TLR4 in this process. The results showed that GP-induced increase in the protein levels of TLR4, MyD88, and TRAF6 was markedly attenuated in the presence of TAK-242 (*p* < 0.01).

We analyzed the phosphorylation status of NF-κB p65 to explore the regulatory function of NF-κB in GP-mediated immune responses. GP significantly upregulated both p65 expression and phosphorylation (p-p65), suggesting NF-κB activation (*p* < 0.01) (Figure 7B1,B2). However, when cells were co-treated with PDTC, p-p65 levels were notably suppressed (*p* < 0.01), suggesting that the inhibitory effect of PDTC on GP-triggered NF-κB signaling. These findings underscore the critical role of NF-κB activation in the immunomodulatory properties of GP and suggest that its inhibition can attenuate the downstream effects triggered by TLR4 activation.

### 3.8. Facilitation of the Nuclear Translocation of NF-κB p65 by GP

**Figure 8:**
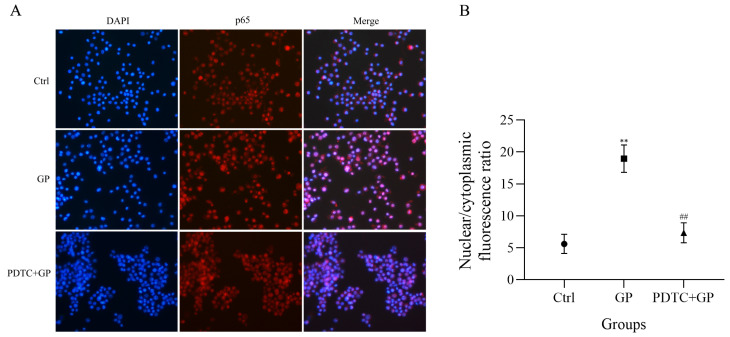
(**A**) The effect of GP on the immunofluorescence analysis of the nuclear translocation of NF-κB p65 in RAW264.7 macrophages. NF-κB p65 is stained red, while cell nuclei are stained blue. (**B**) A quantitative analysis of the nuclear translocation of NF-κB p65. Data represent three independent experiments. ** indicates *p* < 0.01 versus the Ctrl group, while ## signifies *p* < 0.01 for the PDTC + GP group compared to the GP group.

We conducted immunofluorescence analysis to investigate whether GP affects the nuclear translocation of NF-κB p65. After exposure to GP, we observed a marked increase in the fluorescence signal of p65 in the nucleus compared to untreated cells (Figure 8A). We pretreated cells with PDTC, a specific inhibitor of NF-κB signaling, to explore the underlying mechanism. GP-induced increase in the nuclear fluorescence of p65 was substantially reduced after PDTC pretreatment. This observation suggests that GP activates the NF-κB signaling cascade. The cytoplasmic-to-nuclear translocation of NF-κB p65 is a crucial step for controlling gene expression patterns and affecting downstream immune reactions at the cellular level. Furthermore, quantitative analysis of the subcellular localization of p65 revealed a significant increase in its nuclear/cytoplasmic fluorescence ratio in the GP group compared to the control group (*p* < 0.05, Figure 8B), confirming the nuclear translocation of NF-κB following treatment with GP.

## 4. Discussion

The spleen and thymus, as central immune organs, play critical roles in the generation of immune responses and the synthesis of bioactive substances, playing a pivotal role in immune regulation [22]. The weight indices of immune organs directly reflect the dynamic equilibrium of the body’s immune function [23,24], and immune enhancement is typically associated with an increase in immune organ indices. Experimental data revealed markedly reduced thymic and splenic indices in the MC group compared to NC group, validating that LPS can be successfully used to establish a pathological model of mouse immunosuppression. Notably, animals in the GP pretreatment cohort exhibited substantially enhanced spleen indices compared to MC counterparts. Thymic measurements did not show statistical significance between these groups, suggesting that GP may restore the immune function.

This study employed HE staining to conduct a pathological examination of the intestinal mucosa. As the largest immune defense barrier in mammals, the integrity of the intestinal mucosa directly affects its immune function status [23,25]. The intestinal mucosa in the MC group exhibited typical pathological changes: villus height was significantly atrophied, mucosal thickness was reduced, crypt depth showed dysplasia, and the villus-to-crypt ratio decreased by 48%. Morphological alterations confirmed that LPS led to structural damage to the intestinal mucosa, thereby compromising intestinal immune function. Notably, a recovery trend was observed in key indices of the GP group, evidenced by a villus structure reconstruction rate of 75%. These findings confirm the reparative benefits of GP in addressing LPS-induced immune damage.

LPS not only induces immunosuppression but also triggers oxidative stress. Severe oxidative stress markedly enhances the production of ROS and weakens the enzymatic activities of vital antioxidants, including SOD and glutathione peroxidase (GPx) [26,27]. Dysregulation of key antioxidant systems directly disrupts redox homeostasis, leading to secondary damage to the immune defense mechanism [28]. Notably, in this study, GP significantly reversed LPS-induced decreases in the serum activity of SOD, T-AOC levels, and MDA levels in mice. These experimental findings suggest that GP can ameliorate LPS-induced oxidative damage.

To investigate how GP regulates the innate immune response, this study focused on macrophage polarization and measured the immunomodulatory effects of GP on RAW264.7 macrophages, revealing its dual role in enhancing cytophagocytosis activity and promoting the secretion of pro-inflammatory factors by activating the TLR4/NF-κB pathway. Notably, GP exhibited no cytotoxicity at therapeutically relevant concentrations (≤300 μg/mL), while significantly augmenting macrophage viability and function (Figure 4A), highlighting its therapeutic potential. Consistent with the increase in the phagocytic capacity of macrophages (Figure 4B), it has been shown that plant-derived polysaccharides, such as those derived from *Astragalus membranaceus* and *Panax ginseng*, can modulate macrophage surface receptors to facilitate pathogen recognition [29,30]. However, the unique structural characteristics of GP (with acidic pectin backbone), including its abundant acidic groups, such as galacturonic acid [7], may confer distinct binding affinities to TLR4, thereby optimizing membrane fluidity and glycoprotein reorganization to amplify phagocytic efficiency [31]. This structural specificity likely explains the differential effects of GP compared to other polysaccharides, such as β-glucans from *Pleurotus eryngii*, which primarily activate dectin-1 rather than TLR4 [17]. Future studies on the structure-activity relationship (SAR) are warranted to confirm this hypothesis. Specifically, targeted enzymatic or chemical modifications designed to selectively alter GalA content or methylation in GP fragments, coupled with the quantitative assessment of binding affinity toward receptors like TLR4, can provide mechanistic insights. Identifying the correlation between the detailed structural parameters of defined GalA-rich oligo/polysaccharides with their receptor binding and cellular signaling profiles is crucial for establishing predictive SAR models and understanding the precise structural factors determining the bioactivity of GP.

Beyond enhancing phagocytic activity, GP markedly promoted the production of IL-1β, IL-6, and TNF-α (Figure 6A), hallmark cytokines of M1 macrophage polarization [32,33]. The near complete suppression of cytokine release by PDTC (Figure 6A) underscores the central role of NF-κB as the transcriptional orchestrator of GP-induced immune responses. Interestingly, TLR4 inhibition downregulated the expression of MyD88 and TRAF6 (Figure 7A1,A2), but residual NF-κB activation persisted, suggesting potential crosstalk with alternative pathways, such as TRIF-dependent signaling, or scavenger receptor interactions [34]. This finding also indicates that TLR4 may be only one of the receptors for GP. This partial pathway redundancy is similar to the results of studies using polysaccharides from *Physalis alkekengi* L, where TLR4 blockade could not completely inhibit NF-κB activation [35]. Wu et al. also used polysaccharides from *Tetrastigma hemsleyanum* and reported similar results [36]. Specifically, GP-mediated upregulation of TRAF6 (Figure 7A1) suggests a strong reliance on the MyD88-dependent cascade, a feature potentially linked to its galacturonic-acid-rich domains that enhance TLR4 clustering and downstream signal amplification [37]. It is noteworthy that the expected ligand typically does not upregulate its own receptor. However, in this study, TLR4 expression was upregulated after treatment with GP. This phenomenon may be attributed to the regulatory mechanisms of macrophages or to a positive feedback loop in which GP-induced NF-κB activation promotes the transcription of the TLR4 gene. Similar ligand-induced receptor upregulation has been reported in other contexts [38,39]. We suggest that GP-mediated TLR4 upregulation may contribute to sustained TLR4 signaling, potentially underpinning the observed alterations in the immunomodulatory functions of macrophages. Nevertheless, the precise mechanism requires further studies.

Mechanistically, GP-induced phosphorylation and nuclear translocation of p65 (Figure 7B1,B2 and Figure 8) directly correlate with the transcriptional upregulation of iNOS and pro-inflammatory cytokines (Figure 5B), consistent with the role of NF-κB in innate immunity. The differential effects of TAK-242 and PDTC, wherein TLR4 inhibition reduced but did not completely block p65 activation, suggest that GP may act through auxiliary receptors (e.g., complement receptor 3 or dectin-1 receptor) to fine-tune NF-κB dynamics [40,41]. Such multimodal receptor interactions explain the potent immunostimulatory profile of GP compared to single-receptor-targeting polysaccharides. Furthermore, the structure-activity relationship of GP necessitates more in-depth studies. For example, the chemical structure and conformation of polysaccharides can be modified through sulfation, phosphorylation, carboxymethylation, selenization, methylation, and acetylation to enhance their biological properties or confer novel biological activities [42,43,44].

Although we have previously investigated the structural characteristics of GP in earlier studies [7] and conducted a preliminary study on its immunomodulatory function, the underlying molecular mechanisms remain unclear. Owing to the inherent complexity of polysaccharide structures, research on structure-activity relationships and the mechanisms of action has predominantly been confined to in vitro studies [45,46,47]. In this study, GP was administered to each mouse for the first time. The dual effects of GP in alleviating immunosuppression and oxidative stress were analyzed at the animal level. However, as a limitation, the present study lacked a positive control group receiving a well-established immunomodulatory agent or antioxidant. Due to this limitation, we could not directly compare the efficacy of GP with known effective compounds and precisely measure its relative potency or potential advantages. Future studies incorporating appropriate positive controls are needed to comprehensively assess the therapeutic potential of this polysaccharide. The findings of further experiment in vitro elucidating that GP regulates macrophage polarization through a TLR4/NF-κB-dependent mechanism. These outcomes offer a substantial theoretical framework and empirical support for advancing the use of GP as either an innate immunoregulatory agent or a redox-balancing compound. While this study did not directly evaluate the functional activity of T and B lymphocytes, the current findings propose that GP-activated macrophages may initiate downstream Th1-type cellular immune responses via antigen presentation. These insights provide a crucial theoretical foundation for elucidating the role of GP in adaptive immunity and achieving a comprehensive understanding of its immunomodulatory mechanisms.

## 5. Conclusions

In conclusion, our findings indicated an association between GP and attenuation of LPS-induced immunosuppression in mouse models, characterized by the recovery of immune organ indices and reduced structural damage to the intestinal mucosa. Furthermore, GP normalized serum superoxide dismutase activity and total antioxidant capacity in immune-deficient mice. This modulation of oxidative stress markers indicates the therapeutic potential of GP in mitigating LPS-induced damage. Mechanistically, GP may modulate immune responses by affecting critical regulatory genes associated with the TLR4/NF-κB signaling pathway in immune cells. These insights provide valuable groundwork for exploring how pectin polysaccharides regulate the innate immune response at the molecular level.

## Figures and Tables

**Table 1:** Primers sequence for qPCR.

Genes	Sequences
*iNOS*	Forward: 5′-GTTCTCAGCCCAACAATACAAGA-3′
	Reverse: 5′-GTGGACGGGTCGATGTCAC-3′
*IL-1β*	Forward: 5′-GCAACTGTTCCTGAACTCAACT-3′
	Reverse: 5′-ATCTTTTGGGGTCCGTCAACT-3′
*IL-6*	Forward: 5′-TAGTCCTTCCTACCCCAATTTCC-3′
	Reverse: 5′-TTGGTCCTTAGCCACTCCTTC-3′
*TNF-α*	Forward: 5′-CCCTCACACTCAGATCATCTTCT-3′
	Reverse: 5′-GCTACGACGTGGGCTACAG-3′
*β-actin*	Forward: 5′-GGATGCCACAGGATTCCATAC-3′
	Reverse: 5′-TCACCCACACTGTGCCCATCTA-3′

**Table 2:** The effects of GP on the architecture of the intestinal mucosa in mice.

Group	Villus Height (μm)	Crypt Depth (μm)	Mucosal Layer Thickness (μm)	V/C
NC	445.6 ± 17.9 ^a^	90.6 ± 19.0 ^b^	575.7 ± 50.8 ^a^	5.2 ± 0.6 ^a^
MC	343.2 ± 14.8 ^b^	130.0 ± 12.3 ^a^	460.2 ± 14.5 ^b^	2.7 ± 0.4 ^c^
GP	458.3 ± 1.0 ^a^	119.4 ± 5.7 ^ab^	603.1 ± 26.2 ^a^	3.9 ± 0.2 ^b^

Note: V/C represents the proportional relationship between villus height and crypt depth. Within datasets sharing the same column, different lowercase letters are superscripted to denote significant variations (*p* < 0.05) between values.

## Data Availability

The original contributions presented in the study are included in the article, further inquiries can be directed to the corresponding author.
